# Illuminating spatial pharmacology with in situ drug imaging

**DOI:** 10.1002/ctm2.1024

**Published:** 2022-08-28

**Authors:** Zhengyuan Pang, Li Ye

**Affiliations:** ^1^ Department of Neuroscience The Scripps Research Institute La Jolla CA USA; ^2^ Department of Molecular Medicine The Scripps Research Institute La Jolla CA USA

1

In pharmacology, absorption, distribution, metabolism, excretion (ADME) describes in vivo drug actions. Typically, distribution is measured as organ‐specific drug concentrations, with the notion that drugs could have various tendencies to enter and retain in different organs. Such variability can stem from on‐ and off‐target engagement, chemical property of the drug (size, charge, polarity, etc.), or the property of the organ (lipid content, vasculature, blood‐brain barrier, etc.).^1^ While the inter‐organ difference in drug distribution is well‐recognized, cellular diversity of drug engagement within an organ is not easily accessible with conventional pharmacokinetics (PK) and pharmacodynamics (PD) studies. This knowledge gap is becoming increasingly important as we begin to understand how single‐cell level heterogeneity could significantly affect organ physiology and pathology.^2^

A barrier to tracking drug engagement in vivo across tissue compartments and cell types is the lack of tools to visualize drug molecules in situ. Conventional bulk tissue analysis loses spatial and cellular information. Although it retains spatial information, positron emission tomography generally lacks sufficient resolution to resolve cell type identities.^3^ Fluorescence‐based imaging has been widely used with antibodies or messenger ribonucleic acid (mRNA) probes to visualize endogenous biomolecules, but such tags are too bulky to fit small molecule drugs. To circumvent these barriers, we turned to copper(I)‐catalyzed azide‐alkyne cycloaddition (CuAAC) click reaction. By attaching a small, chemically inert alkyne handle to the drug, one can potentially image the drug with an azide fluorescence label after in vivo administration and subsequent click reactions. Similar strategies have been widely successful in chemoproteomics efforts to profile drug targets in cell culture and tissue lysates.^4^ However, direct in situ drug labeling in mammalian tissue has not been feasible due to potential side reactions and low signal‐to‐noise ratio.

In the recent report by Pang et al.,^5^ we developed Clearing Assisted Tissue click CHemistry (CATCH) by combining tissue clearing with in situ click chemistry. This allowed us to visualize covalent drug binding at subcellular resolution directly. Compatible with mainstream histological methods such as immunostaining and in situ hybridization, CATCH can identify where and which cell types within an organ are quantitatively bound by a specific drug, revealing heterogeneous intraorgan drug engagement.

For example, we found: (1) PF7845 and BIA10‐2474, two fatty acid amide hydrolase (FAAH) inhibitors that primarily targeted neurons in the brain, whereas the monoamine oxidase inhibitor pargyline mostly bound blood vessels. (2) The less specific FAAH inhibitor BIA10‐2474 showed off‐target binding in a small nucleus in the pons. (3) At the sub‐saturating dose, PF7845 binding was constrained by vasculature proximity in the hippocampus. (4) At ascending doses, the monoacylglycerol lipase inhibitor MJN110 spread from the axonal to the soma compartment through off‐target binding to FAAH. These findings demonstrate that CATCH can unveil expected and unexpected drug binding across cell types and tissue compartments in a dose‐dependent manner, which can be used to guide drug candidate optimizations for maximizing therapeutic windows.

We are witnessing the rapid development of single‐cell RNA sequencing, proteomics, and spatial transcriptomics technologies that transform our understanding of cellular heterogeneity across organ systems.^2^, ^6^, ^7^ The spatial information on drug engagement revealed by CATCH can be registered onto the ever‐growing multiomics database,^8^ conceptually paving the way for spatially‐resolved pharmacology, which can bring holistic, unbiased assessment of drug efficacy, and toxicity in vivo.

In recent years, covalent kinase inhibitors targeting Bruton's tyrosine kinase (BTK), epidermal growth factor receptor (EGFR), and mutant Kirsten rat sarcoma virus (KRAS‐G12C) have had tremendous successes in treating hematological malignancies and solid tumors.^9^ Yet, questions remain on improving their efficacy while minimizing toxicity by optimizing tissue selectivity,^10^ especially for the central nervous system. CATCH has the potential to greatly accelerate this process by revealing the drug's cellular engagement in both “wanted” and “unwanted” organs, thereby guiding lead selection and modification.

In conclusion, we foresee CATCH can bridge the gap and bring pharmacology study to the exciting single‐cell multiomics era. The concepts and strategies in CATCH can be broadly utilized in future basic chemical biology research and benefit clinical candidate development.

## CONFLICT OF INTEREST

The authors declare that there is no conflict of interest that could be perceived as prejudicing the impartiality of the research reported. The design, steps, and applications of CATCH are covered in pending patent application materials from the Scripps Research Institute.

## References

[1] Xiong B , Wang Y , Chen Y , et al. Strategies for structural modification of small molecules to improve blood‐brain barrier penetration: a recent perspective. J Med Chem. 2021;64(18):13152‐13173. doi:10.1021/acs.jmedchem.1c00910 34505508

[2] Stuart T , Satija R . Integrative single‐cell analysis. Nat Rev Genet. 2019;20:257‐272. doi:10.1038/s41576-019-0093-7 30696980

[3] Gawne PJ , Man F , Blower PJ , de Rosales TMR . Direct cell radiolabeling for in vivo cell tracking with PET and SPECT imaging. Chem Rev. 2022;122:10266‐10318. doi:10.1021/acs.chemrev.1c00767 35549242 PMC9185691

[4] Parker CG , Pratt MR . Click chemistry in proteomic investigations. Cell. 2020;180:605‐632. doi:10.1016/j.cell.2020.01.025 32059777 PMC7087397

[5] Pang Z , Schafroth MA , Ogasawara D , et al. In situ identification of cellular drug targets in mammalian tissue. Cell. 2022;185:1793‐1805. doi:10.1016/j.cell.2022.03.040 35483372 PMC9106931

[6] Kelly RT . Single‐cell proteomics: progress and prospects. Mol Cell Proteomics. 2020;19:1739‐1748. doi:10.1074/mcp.R120.002234 32847821 PMC7664119

[7] Rao A , Barkley D , Franca GS , Yanai I . Exploring tissue architecture using spatial transcriptomics. Nature. 2021;596:211‐220. doi:10.1038/s41586-021-03634-9 34381231 PMC8475179

[8] Subramanian I , Verma S , Kumar S , Jere A , Anamika K . Multi‐omics data integration, interpretation, and its application. Bioinform Biol Insights. 2020;14:1177932219899051. doi:10.1177/1177932219899051 32076369 PMC7003173

[9] Vita ED . 10 years into the resurgence of covalent drugs. Future Med Chem. 2021;13(2):193‐210. 33275063 10.4155/fmc-2020-0236 PMC8356679

[10] Rotow J , Bivona TG . Understanding and targeting resistance mechanisms in NSCLC. Nat Rev Cancer. 2017;17:637‐658. doi:10.1038/nrc.2017.84 29068003

